# Targeting Multiple Signal Transduction Pathways of SARS-CoV-2: Approaches to COVID-19 Therapeutic Candidates

**DOI:** 10.3390/molecules26102917

**Published:** 2021-05-14

**Authors:** Sajad Fakhri, Zeinab Nouri, Seyed Zachariah Moradi, Esra Küpeli Akkol, Sana Piri, Eduardo Sobarzo-Sánchez, Mohammad Hosein Farzaei, Javier Echeverría

**Affiliations:** 1 Pharmaceutical Sciences Research Center, Health Institute, Kermanshah University of Medical Sciences, Kermanshah 6734667149, Iran; pharmacy.sajad@yahoo.com (S.F.); zmoradi@kums.ac.ir (S.Z.M.); sanapiri@ymail.com (S.P.); 2 Student Research Committee, Kermanshah University of Medical Sciences, Kermanshah 6714415153, Iran; zeinab7641@yahoo.com; 3 Medical Biology Research Center, Health Technology Institute, Kermanshah University of Medical Sciences, Kermanshah 6734667149, Iran; 4 Department of Pharmacognosy, Faculty of Pharmacy, Gazi University, Etiler, Ankara 06330, Turkey; esrak@gazi.edu.tr; 5 Instituto de Investigación y Postgrado, Facultad de Ciencias de la Salud, Universidad Central de Chile, Santiago 8330507, Chile; 6 Department of Organic Chemistry, Faculty of Pharmacy, University of Santiago de Compostela, 15782 Santiago de Compostela, Spain; 7 Medical Technology Research Center, Health Technology Institute, Kermanshah University of Medical Sciences, Kermanshah 6734667149, Iran; 8 Departamento de Ciencias del Ambiente, Facultad de Química y Biología, Universidad de Santiago de Chile, Santiago 9170022, Chile

**Keywords:** coronavirus, SARS-CoV-2, COVID-19, signaling pathway, inflammation, oxidative stress, apoptosis, autophagy, natural products

## Abstract

Due to the complicated pathogenic pathways of coronavirus disease 2019 (COVID-19), related medicinal therapies have remained a clinical challenge. COVID-19 highlights the urgent need to develop mechanistic pathogenic pathways and effective agents for preventing/treating future epidemics. As a result, the destructive pathways of COVID-19 are in the line with clinical symptoms induced by severe acute coronary syndrome (SARS), including lung failure and pneumonia. Accordingly, revealing the exact signaling pathways, including inflammation, oxidative stress, apoptosis, and autophagy, as well as relative representative mediators such as tumor necrosis factor-α (TNF-α), nuclear factor erythroid 2-related factor 2 (Nrf2), Bax/caspases, and Beclin/LC3, respectively, will pave the road for combating COVID-19. Prevailing host factors and multiple steps of SARS-CoV-2 attachment/entry, replication, and assembly/release would be hopeful strategies against COVID-19. This is a comprehensive review of the destructive signaling pathways and host–pathogen interaction of SARS-CoV-2, as well as related therapeutic targets and treatment strategies, including potential natural products-based candidates.

## 1. Introduction

As a global pandemic, an outbreak of novel coronavirus, named severe acute respiratory syndrome coronavirus 2 (SARS-CoV-2), caused the coronavirus disease 2019 (COVID-19). It has been a serious leading cause of morbidity and mortality worldwide [1,2]. Coronaviruses (CoVs) are a group of single-stranded enveloped ribonucleic acid viruses which are classified into four genera, including α, β, γ, and δ. In the past two decades, two members of the β-CoVs with a zoonotic origin, including SARS-CoV and Middle East Respiratory Syndrome (MERS)-CoV, caused two epidemics in China during 2002–2003 and in the Middle East in 2012, respectively [3,4,5].

The clinical manifestations of COVID-19 mainly encompass pneumonia-related symptoms, such as fever, cough, and shortness of breath [6]. In severe patients, it would lead to acute respiratory distress syndrome (ARDS), cardiovascular, neurological, hepatic, renal, and gastrointestinal complications [7,8], which all seemed to be correlated with dysregulated mechanisms. It has been well-established that the SARS-CoV-2 genome is closely related to the first SARS-CoV [9]. The underlying mechanisms by which SARS-CoV-2 elicits its detrimental effects remained unclear; however, possible mechanisms encompass inflammation, oxidative stress, apoptosis, autophagy, and the processes associated with virus entry into host cells, such as the endocytic pathway and angiotensin-converting enzyme 2 (ACE2) pathway [8,10,11,12]. As of yet, no specific antiviral drug has been discovered for SARS-CoV-2; hence, extensive studies have been ignited to find effective drugs for targeting the aforementioned pathways and for combating COVID-19. In light of the outbreak, several non-specific medications have been exploited, such as broad-spectrum antiviral, anti-inflammatory, antioxidant, and antiapoptotic therapies, immunotherapeutic agents, antibiotics, and supportive care such as supplementary oxygen [13,14,15]. Despite advancements in providing antiviral drugs, their associated toxicity and high financial costs are a significant hurdle in their clinical applications [16]. Therefore, there exists a dire need to discover new, safe, and more efficacious treatment alternatives to achieve successful healing therapies. 

In recent reviews, the role of oxidative stress [17], inflammation [18,19], and some host factors [20] were developed separately, with no focus on all the therapeutic agents, therapeutic targets, host–pathogen interaction, and dysregulated signaling pathways involved in the pathogenesis of SARS-CoV-2. In the present review, we describe the dysregulated signaling pathways and host–pathogen interaction of SARS-CoV-2, as well as relative therapeutic targets and treatment strategies, concentrating on oxidative stress, inflammation, apoptosis, autophagy, the immune system, and virus life cycle.

## 2. COVID-19: Genetics and Structure 

SARS-CoV-2 is a single-stranded enveloped ribonucleic acid virus with a genome size of 29,903 nucleotides [21]. This single strand of RNA is covered by a phosphorylated capsid protein which, together, both form a nucleocapsid. Phospholipid bilayers shield the nucleocapsid and are coated by spike glycoprotein and, probably, hemagglutinin-esterase protein [22]. The virus genome is comprised of two untranslated regions (UTRs) at the 5′ and 3′ ends, which constitute 265 and 358 nucleotides, respectively, as well as 11 open reading frames (ORFs) that encode 27 structural, non-structural, as well as accessory proteins [23,24]. Two overlapping ORF 1a and 1b contain two-thirds of the genome and encode 16 non-structural proteins (NSPs) within the *pp1ab* gene. These proteins comprise NSP3 (papain-like), NSP5 (3C-like protease domain), NSP12 (RNA-dependent RNA polymerase), NSP13 (helicase), NSP14 (3′–5′ exonuclease), as well as other NSPs that are engaged with the transcription and replication of the viral genome [25,26]. The remaining ORFs code structural proteins including spike protein (S), envelopes protein (E), membrane protein (M), as well as nucleocapsid protein (N), and at least six accessory proteins such as orf3a, orf6, orf7a, orf7b, orf8, and orf10 [27]. In some cases, the *hemagglutinin esterase* gene proposed to increase the virus entry mediated to S protein has been located between ORF1b and ORF S [28]. 

## 3. Clinical Features of COVID-19 Disease 

Most patients infected with SARS-CoV-2 exhibit respiratory complications such as pneumonia and ARDS. ARDS is a common leading cause of death in patients with COVID-19, which is characterized by pulmonary and interstitial tissue devastation [29]. The genetic material of SARS-CoV-2 has been identified in cerebrospinal fluid, indicating that SARS-CoV-2 can directly attack the central nervous system, which contributes to several neurological damages. This procedure develops neurological complications, including headache, encephalitis, impaired consciousness, epilepsy, taste/smell disorders, and nausea/vomiting [30,31,32]. SARS-CoV-2 exploits neuronal pathways, such as the olfactory pathway and blood circulation pathway, to enter the nervous system [33,34,35]. During SARS-CoV-2 infection, impairment of the respiratory gaseous exchange leads to hypoxia and anaerobic metabolism, as well as acidic conditions in the brain, which, in turn, participate in cerebral edema and occlusion of the cerebral circulation, and subsequently lead to headache and acute cerebrovascular disease [36]. SARS-CoV-2 also exerts its deleterious effects on the nervous system through an intracranial cytokine storm [37]. Activation of macrophages, microglia, and astrocytes enhances the release of pro-inflammatory cytokines and provokes nerve degeneration and the apoptotic death of neuronal cells [38]. A growing number of patients infected with SARS-CoV-2 manifest signs or symptoms of liver dysfunction that can be attributed to the respiratory distress syndrome-induced hypoxia and the release of huge circulating detrimental inflammatory mediators with the ability to invade liver cells, causing hepatocyte damage and elevated liver enzymes [39]. Additionally, the downregulation of ACE2 by SARS-CoV-2 may enhance blood pressure, and thereby elevate the risk of intracranial hemorrhage [40]. 

A growing number of patients infected with SARS-CoV-2 manifested signs of liver dysfunction that can be attributed to the respiratory distress syndrome-induced hypoxia and the release of huge circulating detrimental inflammatory mediators with the ability to invade liver cells, causing hepatocyte damages and elevated liver enzymes [39]. 

Cardiovascular complications such as acute myocardial infarction, venous thromboembolic events, myocarditis, and heart failure may also occur in patients with COVID-19 due to direct virus invasion through ACE2, endothelial dysfunction, hypoxia, excessive inflammatory responses, oxidative stress, elevated level of angiotensin (Ag) II, and atherosclerotic plaque rupture [41,42]. Besides, elevated cardiac biomarkers, including troponin T, have also been demonstrated to be associated with increased inflammatory markers, suggesting that myocardial injury is linked to inflammation [43]. 

Gastrointestinal symptoms, such as vomiting, diarrhea, or abdominal pain, are other common clinical manifestations of COVID-19 during the early phases of the disease. Intestinal dysfunction leads to changes in intestinal microbes, thereby promoting inflammatory cytokines [44]. Consequently, ACE2 is highly expressed in the gastrointestinal tract and SARS-CoV-2 directly invades the gut tract through binding with ACE2 receptors. In this regard, ACE2 is considered a key regulator of intestinal inflammation and can enhance the risk of colitis and other gastrointestinal symptoms [45]. 

Clinical data have demonstrated the presence of SARS-CoV-2 particles in urine samples of patients infected with COVID-19 [46]. It has been reported that SARS-CoV-2 possesses detrimental impacts on kidney function and causes signs/symptoms of acute kidney injury [47]. The expression of ACE2 on podocytes and tubule epithelial cells makes the kidney a host candidate for SARS-CoV-2 [48]. Local inflammatory/immune reaction, direct cytotoxic viral effect, hypoxia, as well as secondary infections/sepsis induce the occurrence of endothelial dysfunction, tubular injury, and heavy proteinuria [49]. Therefore, given the involvement of host factors such as ACE and related complications attributed to oxidative stress, as well as inflammation, apoptosis, and autophagy in the complications of COVID-19, targeting them is of great importance.

## 4. SARS-CoV-2 Infection

SARS-CoV-2 undergoes various steps of fusion, uncoating, nucleic acid synthesis, integration, protease, and assembly/release towards infection; therefore, detailed knowledge of infection pathways is critical to tackling COVID-19. It has been well-established that SARS-CoV-2 enters the host cells via two pathways, including the endocytic pathway and non-endosomal pathway, with the help of proteases (e.g., TMPRSS2); both contribute to the release of the nucleocapsid into the cytoplasm [22]. Among those factors, SARS-CoV-2 utilizes the endocytic pathway as the principal mechanism for viral entry into several types of host cells. The S protein on the surface of a coronavirus can interact with the receptor and then invade the host cells through clathrin-mediated endocytosis [11]. Recent advances have highlighted the critical role of such host receptors, including ACE2, glucose-regulated protein 78 (GRP78), cluster of differentiation 147 (CD147), and dipeptidyl peptidase (DPP4) in viral infection. 

### 4.1. ACE2 

Revealing the first phase of viral entry into the host cells, fusion/entry through facilitating co-receptors could be targeted by appropriate therapeutic agents [50]. Recent advances have highlighted the critical role of such host receptors in viral infection, including ErbB1, tyrosine kinase receptors (TKRs) [51], toll-like receptors (TLRs) [52], TNF-α, ILs, interferon (IFN)-γ, and other receptors affecting the immune system [53]. The involvement of other receptors related to T cells has also been shown to play critical roles in viral infection, such as cytotoxic T-lymphocyte antigen 4 (CTLA-4), programmed death1 (PD-1), as well as T-cell immunoglobulin (Ig) and mucin domain-containing molecule 3 (TIM-3) [54,55]. 

Continuous uncoating and nucleic acid synthesis with the involved enzymes of RNA polymerase are other steps in virus replication, including for SARS-CoV-2. Viral chain terminase and proteases have also been shown to be promising targets against COVID-19 complications. As the final step of viral infection, the viral release could also be a hopeful target in combating COVID-19. Nowadays, host co-receptors have been considered critical agents with undeniable roles in stimulating the immune system and increasing viral infection [56]. The analysis of nucleic acid sequence within the spike proteins of SARS-CoV-2 predicted the role of ACE2 in the cellular entry of the virus, which was confirmed by an in vitro study [56]. 

Synthesized ACE2 is folded and N-glycosylated in the endoplasmic reticulum (ER) then passes to Golgi apparatus for further modifications and packaging and is then transported to the plasma membrane [57]. Cleavage of ACE2 by A disintegrin and metalloproteinase 17 (ADAM17) leads to the release of soluble ACE2 into the extracellular environment. Consequently, angiotensin receptor I (AR I) enhances ADAM17 expression which, in turn, elevates soluble ACE2, and can therefore prevent SARS-CoV-2 entrance [57,58]. Additionally, in response to SARS-CoV-2, binding via clathrin-mediated endocytosis and the internalization of both the virus and its receptor, ACE2, occur [59]. 

The rate expression of ACE2 and its cleavage from the cell membrane contribute to the regulation of ACE2 activity [60]. It has been well-established that Ag II, which mitigates ACE2 expression, passes through type II alveolar (AT2) and type I alveolar (AT1)-extracellular-regulated kinase (ERK)/p38 mitogen-activated protein kinase (MAPK) pathway, thereby playing a pivotal role in the regulation of associated receptors [61]. Additionally, hypoxia-induced factor-1α (HIF-1α) enhances the production of ACE, which, in turn, boosts the production of Ag II, and then leads to a reduced level of ACE2 [62]. SARS-CoV-2-induced downregulation of ACE2 leads to an augmentation of the pro-inflammatory factor, Ag II, and causes lung injury [63]. The recognized receptor of SARS-CoV-2, ACE2, is mainly expressed in a small subset of lung cells [64]. Only minimal percentages of monocytes/macrophages in the lung expressed ACE2 [64]. It presents the possibility of direct cellular infection (with no ACE2 engagement) or the existence of other receptors involved in SARS-CoV-2 entrances [65,66]. In general, the critical role of the renin–angiotensin system (RAS) has been indicated in various pathological and physiological processes. Consequently, angiotensinogen is converted to Ag I by renin. Ag I is, in turn, converted to Ag II then to Ag (1–7), and Mas by ACE1 and ACE2, respectively. While Ag II binds to AR I and makes pathological outcomes, Mas binds to MasR to exert protective responses against COVID-19 [67,68]. Therefore, ACE2 could play the double-edged role of being a co-receptor for SARS-CoV-2 entry and generating Mas for protection [69]. As attained by COVID-19 clinical trials, susceptibility to COVID-19 infection is in a direct correlation with the activity of ACE2. Since this enzyme is enriched in the lungs, heart, brain, kidneys, intestine, testes, and placenta [70,71,72], there is a higher rate of virus presence and pathogenesis [73]. These results indicated that Ag II is likely to be the primary target of SARS-CoV-2 in the lungs [68]. Moreover, there are sex differences in the expression of ACE2. Sex hormones in males made a higher expression of ACE2 than in females, with a greater infectious rate [68,74]. The ACE/ACE2 activity ratio in male serum is higher than in females. Individuals with coexisting disorders, including pneumonia [73], diabetes [75], along with aging [74,76,77], cigarette use [78], pregnancy [71,79], hypoxia, and HIF-1α [62,80], were shown to be more susceptible to the dysregulation of the ACE/ACE2 ratio. Overall, the molecular mechanisms and signaling pathways by which SARS-CoV-2 elicits its harmful effects are incompletely understood, and a few molecules have been identified as a target of SARS-CoV-2. For instance, it has been shown that SARS-CoV-2 reinforces chemokine-associated inflammation and fibrosis through IFN, with ACE2-induced Ras/Raf/mitogen-activated protein kinase kinase (MEK)/ERK/ activating protein 1 (AP1) and casein kinase (CK)2- p21-activated kinase 1 (PAK1) signaling pathways [81]. It has been reported that the aforementioned pathway offers the potential for pulmonary vascular remodeling and exaggerated hypoxia [82]. Aberrant activation of PAK1 hinders immune systems and participates in the promotion of viral infection [83]. Therefore, the suppression of PAK1 or it is upstream potentially repressed SARS-CoV-2 infection. In cases of SARS-CoV-2 infection, ACE2 has attracted substantial attention in COVID-19 pathogenicity [69]. Inappropriate regulation of ACE2/Ag (1–7)/Mas receptor and ACE1/Ag II type 1 receptor pathways could enhance ACE2, and thereby increase the chances of viral entry [69,84]. On the other hand, downregulation of ACE2 by SARS-CoV-2 infection inhibits the degradation of Ag II into Ag (1–7), exacerbates inflammation, and leads to vascular permeability and cardiovascular complications [69].

### 4.2. TMPRSS2

It has been well-established that the proteolytic cleavage of the viral envelope glycoprotein by either intracellular or extracellular proteases, such as trypsin, furin, cathepsin, or transmembrane protease serine 2 (TMPRSS2), plays an important role in SARS-CoV entry [85]. Among them, TMPRSS2 has been shown to activate the spike-protein of COVID-19 for viral fusion and infectivity [86]. An accumulation of findings highlighted that the host protease TMPRSS2, employed for the entry of SARS-CoV-2 into lung epithelium, is an attractive target for pharmacologic intervention. It has been shown that pharmacologic inhibition of TMPRSS2 blocks SARS-CoV-2 entry into human lung cells. Additionally, inhibition of TMPRSS2 prevented SARS-CoV-1 infection in animal models. The TMPRSS2 gene expresses a protein of 492 amino acids which anchors to the plasma membrane. It can be divided into the catalytic chain and noncatalytic chain parts through autocatalytic cleavage between Arg255 and Ile256. After cleavage, the majority of mature proteases are membrane-bound, but their substantial portions can be released into the extracellular space [87]. It has been revealed that TMPRSS2 gene promoter possesses 15-bp androgen response element, and TMPRSS2 transcription is upregulated in the presence of androgens [88]. The activation of SARS-CoV by TMPRSS2 suppresses the blockage of SARS-CoV by IFN-induced transmembrane proteins, a class of IFN-stimulated host cell proteins that participate in inhibiting the entry of various enveloped viruses [89]. 

TMPRSS2 is known as a key gene in prostate cancer [90]. The hepatocyte growth factor (HGF)/c-Met cell is activated by TMPRSS2, provoking the survival pathway of HGF/c-Met receptor tyrosine kinase signaling and stimulating a pro-invasive role in prostate cancer cells. TMPRSS2 also induces inflammation by proteolytically activating the protease-activated receptor-2 (PAR-2) in the prostate. Additionally, the upregulation of PAR-2 promotes matrix metalloproteinase-2 (MMP-2) and MMP-9, both of which play a key role in the metastasis of tumor cells [89,91]. 

### 4.3. Glucose-Regulated Protein 78 (GRP78)

Glucose-regulated protein 78 (GRP78), which belongs to the heat shock protein 70 family, is the master chaperone protein present in the lumen of the ER [92,93]. Under cell stress, overexpressed GRP78 can escape ER retention and translocate to the cell membrane [94]. Once localized in the plasma membrane, GRP78 is susceptible to virus recognition, thereby facilitating the viral entry to the host cells. It has been reported that GRP78 is a target receptor of the MERS-CoV spike protein and bat coronavirus HKU9 (bCoV-HKU9) [95]. Recently, the existence of a SARS-CoV-2 spike protein-GRP78 binding site has been predicted using the computational method [96], thus paving the route to design suitable inhibitors to prevent binding and infection. 

### 4.4. The Cluster of Differentiation 147 (CD147)

The cluster of differentiation 147 (CD147), also known as extracellular matrix metalloproteinase inducer, has recently emerged as an important receptor for SARS-CoV-2 [97]. CD147 possesses the ability to interact with various extracellular and intracellular partners which play a key role in the infection process of the human immunodeficiency virus (HIV), measles, and SARS-CoV [98,99]. It has been reported that CD147 can bind with multiple ligands, including cyclophilins, monocarboxylate transporters, caveolin-1, and integrins [100]. As extracellular interactive partners, cyclophilins A and B can bind to CD147 and activate it, thereby increasing the chance of infection of CD147-expressing cells [101]. It has been reported that cyclophilins A and B can interact with nsp1 of SARS-CoV [98]; however, it is yet not understood whether cyclophilins can bind to SARS-CoV-2. In an in vitro study, Wang et al. [102] revealed that meplazumab, an anti-CD147 antibody, significantly hindered the invasion of host cells by SARS-CoV-2. Surprisingly, this report has been supported by a clinical trial in which the anti-CD147 antibody inhibited SARS-CoV-2 spike protein binding and subsequently facilitated a viral clearance [103]. CD147 also participated in the regulation of nuclear factor-kappa B (NF-κB). Moreover, upregulation of CD147 leads to the activation of NF-κB which, in turn, involves inflammation and proliferative responses [104]. Additionally, cyclophilin–CD147 interaction can recruit the immune cells to the sites of inflammation via chemokine-like activity [105]. Cyclophilin 60 is identified as an important contributor protein in the expression and translocation of CD147 to the cell surface [106]. Several other proteins which bind to CD147 may affect its localization. For instance, the interaction of CD147 with the proton-coupled transporters of monocarboxylate, including MCT1 and MCT4 in the cell membrane, is highly dependent on glutamic acid residue 218 in the CD147 transmembrane domain. However, the mutation of this glutamic acid prevents the access of both CD147 and MCT to the cell membrane [107]. It has been also reported that caveolin-1 binds to CD147 on a cell surface, through which it plays a key role in the regulation of clustering and activity of CD147 [108]. As an interacting partner of CD147, integrin β1 interacts with CD147 to regulate integrin-dependent signaling and focal adhesion kinase (FAK) activation, leading to ignition of the downstream signaling Rac/Ras/Raf/ERK and phosphoinositide 3-kinases (PI3K)/Akt pathways and an increase in the metastatic potential of hepatocellular carcinoma [109]. It has been demonstrated that CD147 increases MMPs expression through several signaling pathways, including Janus kinase (JAK)/signal transducer and activator of transcription (STAT), Ras-MEK1-MAPK, and PI3K/Akt signaling pathway [110].

### 4.5. Dipeptidyl Peptidase (DPP4)

Dipeptidyl peptidase (DPP4), also known as CD26, was considered as the main entry receptor for MERS-CoV [111]. The S protein of MERS-CoV specifically interacts with DPP4 receptors, thereby inducing proteolytic activation of viral entrance and viral membrane fusion with the cell membrane [112]. There is about an 80% genome sequence similarity between MERS-CoV and SARS-CoV with SARS-CoV-2. Recent evidence has shown that DPP4/CD26 can also bind to the S1 domain of the SARS-CoV-2 spike glycoprotein, indicating the potential role of DPP4/CD26 in SARS-CoV-2 adhesion/virulence [113]. The potential interaction between SARS-CoV-2 spike glycoproteins and DPP4 has been demonstrated by docking studies and needs in-depth clarification in experimental models [114]. Intriguingly, there is also evidence suggesting that DPP4 is implicated in the induction of cytokine storm, oxidative stress, the immune system, and apoptosis [115]. DPP4 has been widely studied because of its proteolytic activity on various cytokines and peptides that participate in different medical conditions [116]. In the case of proteolytic activity, DPP4 reduces incretins such as glucagon-like peptide 1 (GLP-1) and glucose-dependent insulinotropic polypeptide (GIP), subsequently leading to a declined insulin secretion and abnormal glucose level [116]. Additionally, DPP4 proteolysis leads to partial or total alteration in signaling and functionality of its substrates, including peptide tyrosine-tyrosine (PYY), neuropeptide Y (NPY), and stromal-derived factor 1 (e.g., SDF-1 and CXCL12) [117]. Intriguingly, there is also evidence suggesting that DPP4 is implicated in the induction of cytokine storm, activation of NF-κB pathway, oxidative stress, the immune system, and apoptosis [115]. It has been revealed that CD26/DPP4 possesses the ability to directly trigger T cell activation through CARMA1-mediated NF-κB activation in T cells which, in turn, leads to T cell proliferation and pro-inflammatory interleukin (IL)-2 cytokine production [118]. People with diabetes are at higher risk of developing the serious clinical events caused by COVID-19 because chronic hyperglycemia and inflammation contribute to an ineffective immune response [119]. In this line, DPP4 inhibitors and/or GLP-1 receptor analogs are widely used for the control of hyperglycemia in type 2 diabetes [120]. The potential role of DPP4 inhibitors in COVID-19- infected patients with type 2 diabetes is not completely clarified. However, DPP4 may illustrate a potential target for decreasing the progression of the complications of type 2 diabetes in those infected with COVID-19 [119]. Therefore, DPP4 inhibition may hinder the infection and/or development of the COVID-19. 

## 5. COVID-19: Pathogenesis, Dysregulated Pathways and Beyond

Patients infected with SARS-CoV-2 exhibited various clinical manifestations such as fever, dyspnea, myalgia, and viral pneumonia [121]. In complicated patients, ARDS, acute kidney injury, cardiovascular complications, neurological side effects, and multiple organ failure have also been shown to be associated with increased mortality [49,122,123]. While the pathobiology of SARS-CoV-2 and molecular mechanisms behind the aforementioned clinical manifestations are not yet entirely known, the roles of inflammation, oxidative stress, apoptosis, and autophagy are undeniable. 

### 5.1. Role of Inflammation in COVID-19

As previously mentioned, inflammatory pathways play important roles in the highly inflammatory conditions of pathogenesis in COVID-19 [124]. As such, in severe cases of COVID-19, patients showed higher serum levels of inflammatory cytokines, including TNF-α, IL-2, IL-6, IL-7, IL-10, IFN-γ, IL-1β, IL-12, IL-18, IL-33, tumor growth factor-β (TGF-β), macrophage inflammatory protein-1α (MIP-1α), monocyte chemoattractant protein-1 (MCP-1), granulocyte-colony stimulating factor (G-CSF), interferon-inducible protein-10 (IP-10), chemokines (e.g., CXCL8, CXCL9, CXCL10, CCL2, CCL3, CCL5) [13,125,126,127,128], and c-reactive protein (CRP) [129,130,131] in the early phase as major causes of ARDS [132]. Extensive immunological responses, high levels of circulating inflammatory cytokines, substantial lymphopenia, and immune-cell infiltration are closely correlated to immune-pathological changes of targeted organs [133]. 

In COVID-19 patients, increased neutrophils/CRP and decreased lymphocytes were revealed; this was in direct correlation with disease severity [13]. Releasing the aforementioned inflammatory factors is also called a cytokine storm, which, in turn, leads to various pathogenic complications in COVID-19 [134,135,136]. The innate immune system also employs IFN type I, IFN-α and IFN-β, and IFN-stimulated response element (ISRE) as downstream mediators in exerting a critical response against viral infection, while a reduced IFN leads to rapid viral replication [137,138]. Consequently, IFN-α/β suppresses viral dissemination/replication in the early stage of viral infection. COVID-19 employs multiple ways toward interfering with the aforementioned pathways of type I IFN production [127,139], including JAK-STAT/ISRE pathway phosphorylation [140]. Following the production of type I IFN, COVID-19 is equipped to suppress the inflammatory pathways [65,140,141], time-dependently [127]. Additionally, any dysregulation in the pathway leads to neutrophil/monocyte/macrophage activation and lethal pneumonia or acute respiratory distress syndrome [127]. A disturbance in the regulation of IFNs generation of pro-inflammatory cytokines produced by macrophages contributes to the apoptosis of T cells, which further hampers viral elimination [142]. During viral infection and activation of the adaptive immune response, the engagement of the T cell receptor provokes intracellular calcium overload which, in turn, induces calmodulin binding to calcineurin. Calcineurin activation participates in the nuclear factor of activated T-cell (NFAT) dephosphorylation [143]. The calcium-calcineurin-NFAT pathway boosts the generation of pro-inflammatory cytokines, thereby maintaining chronic inflammation conditions [144].

As other involved receptors, TLR-7 and TLR-3 activate the downstream signaling cascade, including NF-κB and IFN regulatory factor 3 (IRF3) [140]. Enhanced levels of pro-inflammatory cytokines and the migration of inflammatory cells into the lung tissues are the postulated mechanisms for acute lung injury. Cytokine storm disrupts tissue integrity and subsequently leads to pneumonitis [145]. Activation of various inflammatory cytokines involved in the cytokine storm is controlled by the intracellular signaling pathway JAK/STAT [146]. For instance, IL-6 which has been proven as a pivotal inflammatory cytokine, employs the JAK/STAT pathway to perform its biological functions such as immune response, inflammation, and oxidative stress. The inhibition of the IL-6/JAK/STAT pathway appears a promising therapeutic option for the alleviation of COVID-19 [147]. 

### 5.2. Role of Oxidative Stress in COVID-19

Oxidative stress is considered a key contributor to the severity and pathogenesis of SARS-CoV-2. Over-generation of reactive oxygen species (ROS) and antioxidant depletion drive a pivotal role in viral replication and viral-related complications [148,149]. Some populations of innate immune cells, such as macrophages and neutrophils, would generate ROS to clear the pathogens [150,151]. Despite the necessity of ROS production by macrophages and monocytes for modulating immune responses and eliminating viral infection, related over-production contributes to the oxidation of cellular proteins/lipids and corrupts both infected and normal cells, thereby leading to multiple organ dysfunctions [152]. Moreover, compelling studies have shown that viral infections such a SARS-CoV are linked to the inhibition of Nrf2 and augmentation of NF-κB signaling, leading to antioxidant deprivation and inflammation [153]. Nrf2, and its downstream target antioxidant enzyme heme oxygenase-1 (HO-1), serves as a crucial signaling pathway for cytoprotection against inflammation through inhibiting critical inflammatory regulatory pathways such as NF-κB [148]. Interestingly, Nrf2-keap1/HO-1 activation accompanied by an increase in enzymatic/non- enzymatic antioxidant activities, including superoxide dismutase (SOD), catalase (CAT), glutathione peroxidase (GPx), glutathione (GSH), thiobarbituric acid reductase (TBARS), NAD(P)H:quinone oxidoreductase 1 (NQO-1), which, in turn, suppress oxidative mediators and lipid peroxidation, thereby alleviating the hallmarks of viral infection [154,155]. Therefore, the Nrf2 pathway is an auspicious therapeutic target for combating SARS-CoV pathogenesis.

### 5.3. Role of Apoptosis in COVID-19

Apoptosis is a determiner pathway involved in COVID-19 complications. As a pathogenic pathway, apoptosis induction in infected cells can directly lead to viral pathogenesis [156]. In SARS-CoV-infected patients, lymphopenia may occur due to T cell diminution through the activation of apoptosis [157]. Apoptosis activation mediated by human COVID-19 infection contributes to the spread of the virus [158]. Apoptosis activation is associated with numerous abnormalities in virally infected organs. In this line, SARS-CoV-2 infection stimulated apoptosis in lung epithelial/endothelial cells, which causes vascular leakage and alveolar edema, as well as acute lung injury [29]. Several mechanisms are involved in apoptosis activation by human COVID-19. It has been reported that human COVID-19 stimulates apoptosis via ER, caspase-mediated, p38MAPK, and c-Jun N-terminal kinase (JNK) dependent pathways, which are needed for viral replication [159,160]. From another point of view, SARS-CoV triggers apoptosis through decreasing anti-apoptotic B-cell lymphoma 2 (Bcl)-2 members (e.g., Bcl-2 and Bcl-xL) and key survival signaling pathways such as Akt. The upregulation of Akt inactivated several pro-apoptotic molecules such as glycogen synthase kinase-3β (GSK-3β), caspase-9, Bad, and forkhead transcription factor Foxo1 (FKHR), thereby hampering apoptotic pathways [161]. Virus infection can trigger poly (ADP ribose) polymerase (PARP) and ultimately result in apoptosis. PARP drives an important role in programmed cell death and cytokine release [162,163]. Therefore, PARP inhibitors can be served as supportive treatments for alleviating the hallmarks of COVID-19. Besides, viral infections disrupt mitochondrial membrane potential and provoke pro-apoptotic factors such as cytochrome C, caspase-9, and caspase-3 [164,165]. Therefore, targeting particular mediators and enzymes of the apoptotic pathway is an attractive strategy for fighting a viral infection.

### 5.4. Role of Autophagy in COVID-19 

As another critical pathway for COVID-19, autophagy is an intracellular regulated process that plays a pivotal role in the maintenance of cellular homeostasis [166]. Considering mechanistic changes in COVID-19, autophagy is a fundamental cell process in the pathogenicity of disease. This process is characterized by the formation of the double-membrane autophagosomes that subsequently fuse with acidic lysosomes to form autolysosomes through a pH-dependent mechanism. The engulfed components are then degraded with lysosomal enzymes [167]. There is increasing evidence that dysregulated autophagy seems to play an essential role in the pathogenesis of SARS-CoV, as well as its arising complications. Altered autophagy caused by viral infection is strongly associated with severe tissue damage. On the other hand, autophagy could be considered a double-edged sword in the pathogenesis of SARS-CoV. The pro-viral or antiviral role of autophagy remains unclear [149]. The virus that enters the host cell can either be eliminated via autophagy or escape autophagic degradation and replicate in the host cell [168]. A central aspect of the pro-viral role of autophagy is to boost viral replication by the formation of double-membrane vesicles in the host cells. In fact, virus replication in the host cell begins at the ER-Golgi intermediate compartment, which is connected to autophagosome biogenesis, where the viral genome possesses a critical interaction with the proteins that are necessary to assemble a complete virus [169,170]. It has been identified that viral nsp6 protein was found to co-localize with the endogenous autophagy marker, LC3, suggesting a possible collaboration between autophagy and COVID-19 replication [168]. Therapeutics such as chloroquine and hydroxychloroquine elicit antiviral effects by inhibiting the fusion of autophagosomes and lysosomes, and blocks the later stages of autophagic flux [171]. On the other hand, the induction of autophagy may combat viral infection by the degradation of viral components and the augmentation of innate and adaptive immunity [172]. Induction of autophagy and inflammatory responses induced by viral infection contribute to lung injury [173]. It has been reported that the inhibition of S-phase kinase-associated protein 2 (SKP2), which is responsible for proteasomal degradation of Beclin 1, enhanced autophagy, and subsequently attenuated the replication of MERS-CoV [174]. A novel analysis has also highlighted the relation between autophagy mechanisms and antiviral/inflammatory responses in COVID-19. In this sense, PI3K/Akt/ mammalian target of rapamycin (mTOR) is a key control signaling pathway for autophagy that regulates various autophagy mediators, such as Beclin, microtuble-associated protein light chain 3 (LC3), and autophagy-related (Atg). Human COVID-19-infected hepatocytes could induce autophagy through ERK/MAPK and inhibition of the PI3K/Akt/mTOR pathway [175]. Additionally, JNK, AMP-activated protein kinase (AMPK), p38MAPK control the balance of the autophagy response to viral infection [176,177,178]. Considering the role of the aforementioned mediators in autophagy, modulating autophagic pathways could pave the road for combating SARS-CoV-2 infection [170]. 

Overall, the inhibition of autophagy during the first phase of COVID-19 could prevent the replication of SARS-CoV-2 and negatively regulate the IFN response. On the other hand, autophagic pathways are in a near link to inflammation and immune responses in COVID-19. Accordingly, dysregulation in autophagic pathways could lead to cytokine storm and immune dysfunction. Consequently, autophagy modulation restores homeostasis in the immune response of COVID-19 to represent an important challenge, indicating the ability to improve antiviral response, restrict inflammation, and prevent other complications [179]. Therefore, a mechanistic targeting of autophagy should be considered a new strategy in combating SARS-CoV-2. 

## 6. Therapeutic Interventions for COVID-19 

Shortly after the identification of COVID-19 in China, many studies demonstrated the effectiveness and advantages of different classes of drugs when hoping to find a suitable agent with promising effects in the prevention, control, recovery, and improvement of related pathological conditions. It has been well-established that inflammation, apoptosis, oxidative stress, autophagy, and host factors, as well as destructive signaling pathways, play a crucial role in the pathogenesis of SARS-CoV-2. Therefore, modulation of the dysregulated therapeutic targets and pathways is an attractive therapeutic avenue for COVID-19. 

### 6.1. Targeting Autophagy and Apoptosis 

Prevailing evidence has highlighted the cross-talk and the balanced interplay between autophagy and apoptosis [180]. The over-accumulation of autophagosome promotes the apoptotic pathway that eventually causes apoptotic death of the virally infected cells and represses the virus replication cycle [181]. Therefore, providing alternative therapies that potentially interfere with SARS-CoV-2 and lead to autophagy regulation is of great importance. To date, there are no proven effective therapies to prevent or cure COVID-19. An accumulation of findings suggests that several drugs under clinical trials for SARS-COV-2 are autophagy/apoptosis modulators. For instance, chloroquine/hydroxychloroquine, emtricitabine/tenofovir, IFN-α-2b, lopinavir/ritonavir, and ruxolitinib contribute to autophagosome accumulation through inhibiting autolysosome formation and thereby disrupt the replication of SARS-CoV-2 [182]. Additionally, corticosteroids suppress autophagy by inhibiting LC3 recruitment [183]. Besides, ruxolitinib, as a JAK inhibitor can induce autophagy through blocking mTORC [184]. 

Altogether, modulating apoptosis and autophagy seems to be a hopeful strategy in combating COVID-19.

### 6.2. Targeting Oxidative Stress 

Viral infections provoke cytokine storm, which in turn leads to oxidative damage. Therefore, alleviation and management of oxidative damages can be achieved by a large dose of antioxidants [185]. Vitamin C possesses well-characterized antioxidant properties, being able to scavenge free radicals and thereby prevent cells and tissues from oxidative damage [186]. Apart from its antioxidant property, evidence is accumulating that vitamin C exhibits antiviral activity by augmenting IFN-α production, decreasing inflammation, ameliorating endothelial dysfunction, and also direct virucidal activity [187]. A randomized placebo-controlled trial revealed that the high dose of intravenous vitamin C can improve pulmonary function and decrease the risk of ARDS in 308 patients diagnosed with COVID-19 and transferred into the intensive care unit [188]. Vitamin E is a key lipophilic antioxidant that mitigates lipid peroxidation [189]. This vitamin also regulates immune response and stabilizes membrane cells. The important effects of vitamin E make it a potential candidate for the alleviation of oxidative damage and inflammation induced by SARS-COV-2 [190]. More importantly, astaxanthin, a lipid-soluble carotenoid that possesses a higher antioxidant effect than vitamin E and vitamin C, can be considered as a potential option in counteracting COVID-19 complications [191]. 

### 6.3. Targeting SARS-CoV-2 Invasion

Targeting the lifecycle steps of SARS-CoV-2, including virus attachment and endocytosis, viral replication, and transcription, as well as virus assembly and release, provides a promising therapeutic approach. The auspicious drug targets encompass host factors (e.g., ACE2, TMPRSS2, and CD147), and NSPs (e.g., RNA-dependent RNA polymerase, and 3-chymotrypsin-like protease), along with structural proteins. Interestingly, serine protease inhibitors, such as camostat, were identified as suppressing TMPRSS2 and effectively decreasing mortality following SARS-CoV infection [11]. More importantly, Hoffmann et al. revealed that this drug possesses the ability to abrogate SARS-CoV-2 entry into lung cells by suppressing ACE2 and TMPRSS2 [192]. Based on preclinical investigations, a double-blind randomized controlled clinical study was performed with 114 COVID-19 infected patients to find whether camostat mesylate at a dose of 200 mg/3 times a day can diminish a SARS-COV-2 viral load in early COVID-19 disease (NCT04353284). In an open-label phase 2 clinical trial, meplazumab, an anti-CD147 antibody, inhibited SARS-CoV-2 spike protein binding and could block the infection of SARS-CoV-2 in 20 COVID-19 patients with pneumonia [103].

It has been reported that arbidol possesses an attractive mechanism of action that affects the S protein/ACE2 interaction, halting viral membrane fusion [193]. A non-randomized study revealed that treatment with arbidol for nine days decreased mortality rates and enhanced discharge rates in 67 patients infected with COVID-19 [194]. As antiviral chances, combined lopinavir/ritonavir combination as 3-chymotrypsin-like protease inhibitors of anti-retroviral drugs was suggested as an effective drug against MERS-CoV and SARS-CoV [195,196]. For this reason, several clinical trials have been performed to investigate its effects on COVID-19. The results of those studies were not sufficient and did not recommend combination therapy as a suitable medication [1,197]. The advantages of new studies emphasized that arbidol monotherapy was more impressive than lopinavir/ritonavir in the treatment of patients with COVID-19. About 14 days after the treatment, viral load was not identified in the arbidol group, and the duration of the positive RNA test was shorter in this group [198].

Favipiravir is a broad-spectrum RNA polymerase inhibitor, an antiviral compound that showed a suitable activity versus the Crimean-Congo hemorrhagic fever, rabies, oseltamivir-resistant, and wild-type influenza B virus in mice [199,200,201]. For this reason, an open-label control study was performed to investigate the advantages of favipiravir on COVID-19, and results showed improvement in the chest imaging in comparison with the control group and might be a useful agent in the treatment of COVID-19 [202]. Moreover, remdesivir is a new nucleotide analog and RNA-dependent polymerase inhibitor that showed considerable in vitro activity versus SARS-CoV-2 [203]. An emergency use authorization for remdesivir was issued to adults and children hospitalized with COVID-19. Wang et al. designed a double-blind, randomized trial to investigate the effect of remdesivir in 237 patients with severe COVID-19. Compared with placebo, remdesivir could not significantly reduce the duration of hospitalized time in patients with COVID-19 [204]. Besides, another RNA-dependent polymerase inhibitor, ribavirin, which is routinely used in combination with IFN for hepatitis C virus infection, could not find enough evidence to treat COVID-19 [205]. Darunavir is a protease inhibitor that has shown beneficial effects in treating HIV-1 infection. In February 2020, a clinical trial was registered in China to peruse the advantages of this drug in combination with cobicistat, a human cytochrome P-450 3A enzyme inhibitor; thus far, no data support the efficacy and safety of this agent in humans diagnosed with COVID-19 (NCT04252274). 

There is inadequate and insufficient information thus far to know whether chloroquine or hydroxychloroquine has a remarkable role in the treatment of COVID-19. Both hydroxychloroquine and chloroquine have been documented to inhibit SARS-CoV-2 in vitro; however, it seems that the antiviral potential of hydroxychloroquine is more than chloroquine. The antiviral mechanisms of hydroxychloroquine and chloroquine are not fully realized, but inhibiting viral fusion, changing the pH at the cell membrane surface, inhibiting and suppressing the replication of nucleic acid, preventing viral assembly and release, and decreasing the glycosylation of viral proteins are amongst their important possible antiviral mechanism [203,206]. Even so, the obtained clinical data on either of the two compounds are limited and have serious methodological problems. In an open-label study performed in March 2020, the administration of 200 mg hydroxychloroquine three times per day for ten days increased the rate of undetectable SARS-CoV-2 RNA in samples obtained from nasopharyngeal in comparison with the placebo group [207]. Significant methodologic problems reduced the value of those studies and made the results unreliable [208]. Another randomized trial with a statistical population of 30 adults with COVID-19 was performed in Shanghai. The results of those studies did not show a significant difference between the group receiving hydroxychloroquine and the group receiving standard care [209]. Furthermore, adverse effects due to high doses of chloroquine and increased mortality prevented patients from continuing the studies [210]. 

### 6.4. Targeting Inflammation 

From an inflammatory point of view, the critical role of inflammatory responses and enhanced inflammatory cytokines in COVID-19 are the most critical factors. In this regard, the level of IL-6 showed a considerable correlation with the severity of COVID-19, and the measure of this cytokine can be used as an important factor in predicting disease severity [211]. Tocilizumab is a selective antagonist of the IL-6 receptor, which prevented cytokine release syndrome and led to improving the conditions of a patient with severe COVID-19 [212]. Several clinical studies have been conducted in various countries, including the United States, Spain, Nepal, Malaysia, and Belgium, to investigate the effects of this drug, but the full results of these studies have not yet been published (NCT04332094, NCT04377659, NCT04331795, NCT04330638, NCT04317092, NCT04345445). Siltuximab and sarilumab are other receptor antagonists of IL-6 that are in the early stages of clinical research (NCT04329650, NCT04322188, NCT04341870, NCT04357808). Consistently, it seems that the IFN-β Subtype may be a suitable option for COVID-19 treatment. IFN-β properly decreased the MERS-CoV in vitro and has had pleasant outcomes in an animal model of MERS-CoV infection, but no data evaluated the advantages of IFN-β on SARS-CoV-2 [195,213,214].

As the importance of JAK/STAT in the pathogenesis of COVID-19 was shown previously, baricitinib is a JAK inhibitor that leads to the inactivation of STATs and a decrease in the serum levels of IgG, IgA, IgM, and CRP. The limited data demonstrated that the administration of baricitinib may modify cytokine-release syndrome due to COVID-19. The results suggested this agent as a useful drug for damascening to the COVID-19 therapy regimen [215]; for this reason, several clinical trials are in progress to sift the effect of baricitinib in COVID-19 (NCT04340232, NCT04321993, NCT04362943).

Glucocorticoids are of the main classes of drugs possessing immunosuppressive, anti-inflammatory, and antiproliferative activities through blocking IL-1α and β, NF-κB, TNF-α, AP-1, and increasing the synthesis of, IκB-α. The administration of glucocorticoids in patients with influenza led to a delay in viral clearance and enhanced risk for mortality; this was similar in patients with a MERS-CoV infection [216,217]. Furthermore, the administration of glucocorticoid drugs on patients with COVID-19 did not provide adequate and acceptable results [218]. Nonsteroidal anti-inflammatory drugs (NSAIDs) have for a long time been considered effective therapies against inflammatory diseases [219]. To determine the efficacy of ibuprofen, a commonly prescribed NSAID, in COVID-19, a randomized phase 4 clinical study was applied in 230 severe COVID-19 patients to treat them with ibuprofen at a daily dose of 200 mg (NCT04334629). Additionally, a randomized phase 3 clinical trial was registered to assess the effectiveness of naproxen (250 mg twice a day) in patients (n = 584) infected with SARS-CoV2 (NCT04325633) [220]. Pre-clinical evidence has previously been presented on the use of NSAIDs during COVID-19 [221]. 

### 6.5. Miscellaneous Agents

In addition to the aforementioned agents, antibiotics are used for possible effectiveness in combating COVID-19. Azithromycin is a macrolide antibiotic with conflicting information about its concomitant use with hydroxychloroquine for COVID-19 treatment. However, a study conducted in May 2020 in France showed that the use of azithromycin in combination with hydroxychloroquine before the beginning of COVID-19 complications may be safe and led to a very low fatality rate in patients [222]. The significant potential of both drugs for corrected QT interval prolongation, as well as the possibility for the exacerbation of this complication in their simultaneous use, prevents their concomitant administration, and it is not recommended [223,224]. As another antibiotic, teicoplanin was shown to be effective against former coronaviruses and demonstrated an in vitro activity against the novel coronavirus, but enough information and convincing evidence are not available from clinical trials [225]. 

As another class of drugs, an anti-parasitic drug, ivermectin, showed a suitable in vitro effect on SARS-CoV-2 [226]. For this reason, the authors advised investigating the possible benefits of ivermectin in humans with COVID-19, and several clinical trials began in the hope of achieving convincing results (NCT04360356, NCT04343092, and NCT04374279). From another point of view, oseltamivir, a neuraminidase inhibitor, indicated for prophylaxis and treatment of influenza, did not show any significant effect for treatment or prophylaxis of COVID-19 [205]. From other drugs used against COVID-19, the Bacillus Calmette–Guérin (BCG) vaccine could be mentioned for its use in the immunization against tuberculosis and the prevention of leprosy. The BCG vaccine showed in vitro and in vivo non-specific protective activities versus other respiratory tract infections. Statistical analysis was conducted to investigate the effects of vaccine BCG in countries with and without national vaccination programs in preventing and reducing COVID-19’s mortality. The results showed that, in countries with vaccination programs, the prevalence and mortality rate was estimated at 38.4 and 4.28 people per million, respectively. The death rate was 40/million in countries without BCG programs [227]. Therefore, it is hypothesized that the vaccine may reduce the incidence and severity of COVID-19 in healthcare workers. In this regard, several clinical trials are being conducted to investigate these effects (NCT04348370, NCT04373291, NCT04327206, NCT04350931, NCT04328441). Increasing evidence has shown that vitamin D deficiency is correlated with COVID-19-associated coagulopathy, inflammation, immune response dysfunction, and mortality [228]. From a mechanistic angle, vitamin D displays antiviral activity through immunomodulation and induction of autophagy [229]. It has been reported that vitamin D supplementation mitigates liver disease progression and augments responses to therapy in hepatitis C virus patients [230].

Other miscellaneous compounds, antioxidation, immune-modulatory, and anti-inflammatory activity of melatonin, as a neurohormone, made this compound one of the drugs with the potential to be added to the therapeutic regimen of patients with COVID-19 [231]. There is some other information on the protective effects of melatonin in viral diseases, which may display these advantages in patients with COVID-19 [231].

## 7. Importance of Phytochemicals in Combating COVID-19

Phytochemicals are a consequential source of active chemicals constructed by plants, with potential effects against pathogens. They have been introduced as influential resources for drug discovery, possessing various human health benefits. Besides, a substantial spectrum of biological activities is reported for phytochemicals, such as anticancer, antibacterial, neuroprotective, cardioprotective, immune-modulatory, anti-inflammatory, and antioxidant effects [232,233]. Several steps in viral replication and infection can be also suppressed by natural products [50]. Although many of these compounds have been shown to have broad-spectrum antiviral effects, the mechanisms behind these effects have not yet been fully elucidated. Besides their potent antioxidant activities, inhibiting the synthesis of DNA and RNA, suitable scavenging capacities, prevention of the virus entry, or reproduction of the virus are some of the critical reported antiviral mechanisms of these compounds [234]. The immune-modulatory effect of natural products and the significant potential for suppressing the inflammatory reaction, as one of the major reasons for mortality and morbidity of SARS-CoV-2 infection, are other promising mechanisms of phytochemicals in the treatment of SARS-CoV-2 [235]. We have previously reported the modulatory roles of natural products on inflammatory, apoptotic, and oxidative stress pathways involved in the pathogenesis of COVID-19-associated lung injury [35,236]. We have also shown that the neuronal manifestations of COVID-19 could be potentially targeted by phytochemicals [35]. Therefore, in the present study, we have focused on the inflammatory, apoptotic, oxidative stress and autophagic pathways, and virus life cycle, as well as the phytochemical effects. Flavonoids, polyphenolics, alkaloids, terpenoids, coumarins, and carotenoids are some of the important groups of phytochemicals with antiviral, antioxidant, and anti-inflammatory activities towards developing a suitable therapeutic option for COVID-19 [35]. 

As a group of multi-targeted agents, flavonoids and polyphenols are already recognized as potential therapeutic agents for the treatment of viral infections. Numerous studies have shown that curcumin (a phenolic compound) possesses anti-inflammatory and antioxidant roles, and interrupts the viral infection process via several mechanisms, including hindering virus entry, replication, and budding, directly interfering with viral proteins and repressing the gene expression of the virus [237,238,239,240]. From another point of view, curcumin reduced the pro-inflammatory cytokines and virus-induced cytokine storm, as well as alleviating lung injury, thereby indicating potential effects in the treatment of COVID-19 [237,241]. According to computational methods, curcumin offers the ability to inhibit the spike protein of SARS-CoV-2 and disrupts viral entry [242]. Another in silico approach also revealed that curcumin and catechin were used as potential antiviral polyphenols through the dual inhibition of host cell receptors to the virus (mediated by ACE2) and viral protein entry (S-protein). It should be mentioned that the binding affinity of catechin was more than that of curcumin [243]. 

It has been previously documented that the bioactive flavonoid baicalein blocks influenza A virus H3N2 through suppressing autophagy markers, Atg-5, Atg-12, and LC3-II [244]. Baicalein could effectively abrogate the replication of SARS-CoV-2 in Vero cells through diminishing 3C-like proteases (3CL^pro^) SARS-CoV-2 [245]. In another study, baicalein mitigated Vero E6 cell damage induced by SARS-CoV-2. Additionally, this nutraceutical agent alleviated the lesions of lung tissue and suppressed replication of SARS-CoV-2 in mice. Furthermore, this compound improved respiratory function and reduced inflammation, corroborated by decreasing the level of IL-1β and TNF-α in lipopolysaccharide (LPS)-induced acute lung injury of mice [246]. Docking evidence revealed that flavonoids biochanin A and silymarin strongly interact with the active site of SARS-CoV-2 spike glycoprotein and ACE2, respectively [247]. 

Further in vitro studies should be carried out on these flavonoids against SARS-CoV-2. As a best-docked bioflavonoid, naringin exhibited a high-affinity binding at the binding site of main protease (M^pro^) and spike glycoprotein of SARS-CoV-2 [248,249]. It has been recently reported that two-pore channel 2 (TPC2) is a key requirement for SARS-CoV-2 entry [250]. Surprisingly, naringenin exhibited the capacity of potent antiviral activity against SARS-CoV-2 and could successfully abrogate TPC2 in vitro [251]. Further in vivo studies are needed to confirm the beneficial effect of naringenin. 

It has been previously documented that phytoactive flavonoid taxifolin ameliorated sepsis-induced pulmonary damage and edema by inhibiting the NF-κB pathway [252]. According to the molecular docking approach, taxifolin was found a potential inhibitor against M^pro^ SARS-CoV-2 [253]. As an effective candidate against SARS-CoV-2, the flavonoid silibinin declined immune response and inflammation by inhibiting STAT3, thereby facilitating effects on the early stage SARS-CoV-2 infection [254]. A computational study proposed that silibinin hinders the replication of SARS-CoV-2 via abrogating RNA-dependent RNA polymerase (RdRp) [255]. Silibinin offers excellent opportunities for further investigations in preclinical and clinical trials as an anti-SARS-CoV-2 agent. It has been reported that the flavonoid luteolin can disrupt the viral fusion and entry process and can also mitigate SARS-CoV infection with EC_50_ values of 10.6 µM in a dose-dependent manner [256]. 

Resveratrol is a phenolic compound that appreciably inhibited MERS-CoV infection and could enhance cellular survival behind virus infection. It could notably decrease an essential protein expression for MERS-CoV replication (nucleocapsid N), and downregulated the in vitro apoptosis induced through MERS-CoV [257]. It has been also shown that resveratrol potentially suppressed SARS-CoV-2 infection in vitro [258]. Emodin is another chemical compound that belongs to the anthraquinone category. It was shown to block and suppress the S protein and ACE2 interaction, leading to beneficial effects in the treatment of SARS-CoV [259]. Hirsutenone, as a bioactive diarylheptanoid polyphenol isolated from *Alnus japonica* (Thunb.) Steud. (Betulaceae), exerted strong antiviral activity through diminishing papain-like protease (PL^pro^) of SARS-CoV. It has been reported that catechol and α, β-unsaturated carbonyl moiety play critical roles in protease blocking activity [260].

Alkaloids are an important class of natural products with antiviral activities, which have been extensively studied [261,262]. Molecular docking evidence proved the promising potential of such alkaloids in targeting SARS-CoV-2 RdRp, including 10′–hydroxyusambarensine, cryptospirolepine, and strychnopentamine [263]. The bioactive alkaloid emetine, as a viral entry inhibitor, has previously been shown to block MERS-CoV-mediated infection [264]. Interestingly, emetine inhibits SARS-CoV-2 replication in vitro, and a synergistic effect between the combination of remdesivir and emetine was observed [265]. As another alkaloid compound, lycorine was strongly able to diminish the spread and replication of human coronavirus OC43 (HCoV-OC43) in a mouse central nervous system [264]. It has also been shown that two alkaloids lycorine and oxysophoridine possess the ability to suppress the replication of SARS-CoV-2 in vitro [266]. Consequently, tylophorine, a natural alkaloid, has shown promising beneficial effects against coronavirus porcine transmissible gastroenteritis virus (TGEV) through suppressing JAK2 mediated NF-κB activation related to the inflammatory response. It also inhibited viral replication by interfering with the viral RNA complex [267]. As broad-spectrum antiviral agents, tylophorine based derivatives also blocked SARS-CoV-2, with EC_50_ values of 2.5–14 nM [268]. Such results indicated the potential of tylophorine as a novel therapeutic intervention for COVID-19 infection.

In addition to phenolic compounds and alkaloids, terpenoids could also contain auspicious natural plant-derived secondary metabolites for combating COVID-19 [269]. As a triterpenoid compound, glycyrrhizin has been successfully applied to mitigate virus-induced inflammatory cascades and viral replication [270,271]. It has been well-established that high-mobility group B1 (HMGB1) protein plays a key role in viral infection and replication [272,273]. Interestingly, an in silico study performed by Bailly et al. revealed that glycyrrhizin is a potential binder of HMG box protein, and could thereby be a promising candidate to be evaluated against COVID-19 [274]. Cumulative evidence has demonstrated that natural coumarin compounds possess antioxidant, antiapoptosis, and anti-inflammatory activities toward antiviral effects. Additionally, these agents effectively disrupt various stages in the virus replication cycle, and could thereby be beneficial agents for tackling SARS-CoV-2 [275]. Regarding coumarins, a recent in silico study revealed that some naturally occurring coumarins, including corymbocoumarin, methylgalbanate, and heraclenol, displayed potential antiviral activity through inhibiting M^pro^ [276]. Molecular docking approaches indicated that natural coumarin compound toddacoumaquinone possesses a significant suppressing ability against M^pro^ of SARS-CoV-2, which is necessary for viral replication [277]. Another in silico study also illustrated that the bioactive coumarin inophyllum A remarkably targets M^pro^ [278]. 

Consequently, of other natural products, carotenoids seem to be of potential interest in targeting various steps of the viral life cycle and host proteins [279]. As one of the most potent/efficient carotenoids, astaxanthin has been a promising source of antioxidation and anti-inflammatory agents, with promising potential to combat viral infections and related complications through targeting several destructive signaling mediators [191]. 

Altogether, several findings revealed that phytochemicals possess the ability to suppress SARS-CoV-2 infection. Unfortunately, almost all of the current evidence focused on the efficacy of phytoactive compounds in silico and in vitro models of COVID-19, and the main antiviral mechanisms remain elusive. Therefore, the beneficial effects of phytochemical against COVID-19 and main mechanisms require in-depth research to be verified by preclinical and clinical studies. Toxicological aspects, pharmacokinetics and pharmacodynamics properties and possible side effects, and structure–activity relationship (SAR) analyses need appropriate assessment. 

**Figure 1:**
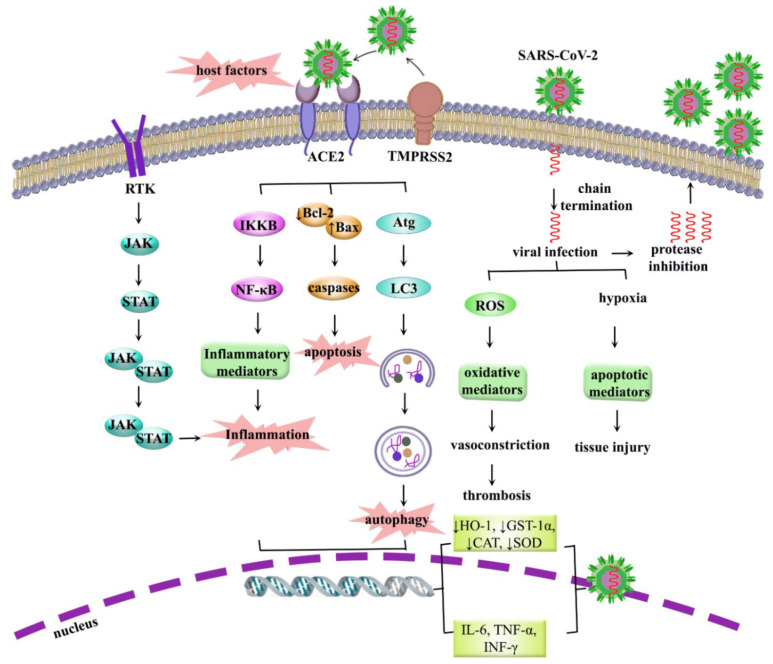
Multiple dysregulated pathways in COVID-19. ACE2: angiotensin-converting enzyme 2; Atg: autophagy related; Bcl-2: B-cell lymphoma 2; CAT: catalase; COX: cyclooxygenase; GST: glutathione S-transferases; HO: heme oxygenase; IFN: interferon; IKKβ: IκB kinase β; IL: interleukin; JAK: Janus kinase; LC3: light chain 3; NF-κB: nuclear factor kappa B; RdRP: RNA-dependent RNA polymerase; RTK: receptor tyrosine kinase; STAT: signal transducer and activator of transcription; TMPRSS2: transmembrane protease serine 2; TNF-α: tumor necrosis factor-α.

In silico studies indicated limonin [280], berberine [281], and fisetin [282] inhibited ACE2 and spike protein [280], bound to ACE2, and increased Nrf2, HO-1, and TGF-β [281]; also led to the reduction of TNF-α, IL-6, IL-1β [282]. Other compounds such as tetrandrine, lycorine, kazinol A [283], and sinigrin [284] inhibited the early stage in HCoV-OC43-infection, and also inhibited the effects against different species of CoV [283], as well as inhibited SARS-CoV 3CL^pro^ and PL^pro^ [283,284]. The results of in silico studies also demonstrated that methyl rosmarinate, calceolarioside B, myricetin 3-*O*-beta-D-glucopyranoside, betulinic acid, cryptotanshinone, dihomo-γ-linolenic acid, kaempferol, quercetin, sugiol, licoleafol, and amaranthine may have striking potential against COVID-19 [285,286]. Based on in silico evidence, different flavonoids, likely tomentin A-E [287], chrysin [288], narcissin [289], cyaniding [290], and hesperetin [291], interacted with ACE2 and declined its neurological manifestation in COVID-19 [288,289,290,291], and also inhibited papain-like protease in COVID-19 [287]. Docking evidence indicated that baicalin binds to TMPRSS2 and leads to the inhibition of COVID-19 [204]. An in vitro study also indicated that geraniol has inhibitory effects against viral spike protein and is a useful agent for therapy against COVID-19 [292]. Additionally, other natural compounds have important roles in modulating those signaling pathways, such as malvidin, which leads to the reduction of Bax/Bcl-2, caspase-3, IL-β, and TNF-α [50]. Additionally, osthole alleviated lung injury and inflammation through preventing the downregulation of ACE2 and Ang1–7 expression, thereby possessing anti-inflammatory effects [293]. Moreover, daidzein reduced TLR4, MyD88, NF-κB, MPO, IL-6, and TNF-α [294], thymol reduced the level of NF-κB, IL-6, TNF-α, and IL-1β [295], hyperin reduced TNF-α, IL-6, IL-1β, and NF-κB [296], and cannabidiol declined the levels of MPO, TNF-α, and IL-6 [297]. These natural products declined the level of important mediators in signaling pathways of COVID-19, and have a vital function in reducing the symptoms of COVID-19. Several phytochemicals with promising antiviral effects are presented in Table 1. Figure 1 shows the proposed targets and related therapeutic candidates for SARS-CoV-2.

ACE2: angiotensin-converting enzyme 2; Bcl-2: B-cell lymphoma 2; COX-2: cyclooxygenase; ERK: extracellular-regulated kinase; GPx: glutathione peroxidase; HCoV: human coronavirus; HO-1: heme oxygenase-1; IL: interleukin; iNOS: inducible nitric oxide synthase; JAK: Janus kinase; JNK: c-Jun N-terminal kinase; M^pro^: main protease; MERS-CoV: Middle East respiratory syndrome coronavirus; MIP: macrophage inflammatory protein; MPO: myeloperoxidase; NF-κB: nuclear factor-kappa B; PL^pro^: papain-like protease; RdRp: RNA-dependent RNA polymerase; Nrf2: nuclear factor erythroid 2-related factor 2; ROS: reactive oxygen species; SARS-CoV-2: severe acute respiratory syndrome coronavirus 2; SOD: superoxide dismutase; TGF-β: tumor grows factor-β; TLRs: toll-like receptors; TNF-α: tumor necrosis factor-α; TPC2: two-pore channel 2.

## 8. Discussion

**Figure 2:**
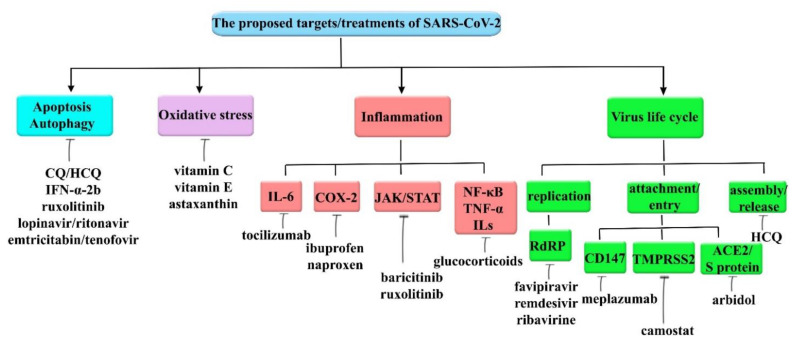
The proposed targets and related therapeutic candidates in SARS-CoV-2. Atg: autophagy-related; CAT: catalase; CQ: chloroquine; HCQ: hydroxyl chloroquine; GST-1 α: glutathione s-transferases-1α; HO-1: heme oxygenase; IFN: interferon; IL: interleukin; JAK/STAT: Janus kinase (JAK)/signal transducer and activator of transcription (STAT); LC3: light chain 3; NF-κB: nuclear factor kappa B; ROS: reactive oxygen species; RTK: receptor tyrosine kinase; SARS-CoV-2: severe acute respiratory syndrome coronavirus 2; SOD: superoxide dismutase; TNF-α: tumor necrosis factor-α.

Due to the complex pathological mechanisms behind COVID-19, revealing its precise signaling pathways may open new roads for providing efficient therapies. COVID-19 employs various signaling pathways/mediators, including inflammation, oxidative stress, apoptotic, and autophagy, to overcome the immune system. It has also been shown to alter the expression of some host factors, including enzymes/mediators and co-receptors such as ACE2, as well as ILs, TNF-α, IFN-γ, Nrf2, Bax/caspases, and Beclin/LC3 to facilitate cellular infection and subsequent complications (Figure 2). Despite advances, medicinal therapy against COVID-19 remains challenging. Besides, considering the multiple mediators involved in the pathogenesis of COVID-19, and providing multi-target agents, could be a more serious step toward controlling an infection. We previously reported the conventional therapeutic agents which potentially target the inflammatory signaling pathways in COVID-19 [124]. The current review introduces candidate therapeutic targets/treatment in COVID-19, as well as the evidence of using candidate phytochemicals. In this regard, phenolic compounds, alkaloids, terpenoids, coumarins, and carotenoids showed potential anti-SARS-CoV-2 effects by targeting viral life cycle, virus entry/replication, spike proteins, ACE2, RdRP, PL^pro^, and M^pro^. It is worth mentioning that, despite preclinical mechanistic studies on the effects of phytochemicals on SARS-CoV-2, more clinical investigations are needed to confirm the results. More studies/methods are also needed to design a novel drug delivery system that counteracts the pharmacokinetic limitations of phytochemicals in COVID-19.

Further areas of research on novel pathophysiological signaling pathways of COVID-19, especially on inflammatory, oxidative stress, apoptotic, and autophagic pathways, will show more potential candidates in the management, prevention, and treatment of COVID-19 complications. That said, more reports are still needed to confirm the benefits of targeting the aforementioned pathways in COVID-19.

## Figures and Tables

**Table 1:** Candidate phytochemicals with promising antiviral effects.

Phytochemical	Compound	Study Type	Mechanism of Antiviral Activity	References
Alkaloid	10′-hydrox-yusambarensine	In silico	↓RdRp	[263]
	Berberine	In vitro, In silico	Antiviral effect, ↓ACE2, spike protein and increased Nrf2, HO-1 ↓TGF-β1, ROS	[281]
	Cryptospirolepine	In silico	↓RdRp	[263]
	Emetin	In vitro	↓Viral entry ↓MERS-CoV S-mediated infection, ↓SARS-CoV-2 replication	[264,265]
	Lycorine	In vivo In vitro	↓Spread and replication of HCoV-OC43, ↓SARS-CoV-2 replication	[264,266]
		In vitro	↓Different species of CoV	[283]
	Oxysophoridine	In vitro	↓SARS-CoV-2 replication	[266,298]
	Strychnopentamine	In silico	↓RdRp	[263]
	Tetrandrine	In vitro	↓HCoV-OC43-infected	[283]
	Tylophorine	In vitro	↓JAK2, ↓NF-κB, ↓inflammation, ↓replication	[267,268]
Anthocyanin	Malvidin	In vitro	↓Bax/Bcl-2, Caspase-3, IL-1β, TNF-α	[50]
Cannabinoid	Cannabidiol	In vitro	↓MPO, TNF-α, IL-6	[297]
Coumarin	Inophyllum A	In silico	↓M^pro^, ↓replication	[278]
	Methylgalbanate	In silico	↓M^pro^, ↓replication	[276]
	Osthole	In vitro	↓IL-6, TNF-α, ↑ACE2 and Ang1–7	[293]
	Toddacoumaquinone	In silico	↓M^pro^, ↓replication	[277]
Diarylheptanoid	Hirsutenone	In vitro	↓PL^pro^, ↓replication	[260]
Flavonoid	Baicalein	In vitro In vivo	↓3CL^pro^ ↓Vero E6 cells damage, ↓lesions of lung tissue, ↓replication, ↓IL-1β, ↓TNF-α, ↓inflammation	[245,246]
	Biochanin A	In silico	↓spike glycoprotein	[247]
	Kaempferol	In vitro In silico	↓3CL^pro^, ↓replication	[299]
	Luteolin	In vitro In silico	↓Viral entry ↓SARS-CoV infection ↓TNF-α, IL-1β, IL-6, IL-18, NF-κB	[256,300]
	Naringenin	In vitro In silico	↓TPC2, ↓viral infection ↓TNF-α, IL-1β, IL-6, IL-18, NF-κB	[251,300]
	Naringin	In silico	↓M^pro^, ↓replication	[249]
		In silico	↓Spike glycoprotein	[248]
	Silibinin	In silico	↓RdRp	[255]
	Silymarin	In silico	↓ACE2 ↓IL-6, IL-1β, TNF-α, p46-p54, p42, p38, p44, NF-κB, and JNK.	[247]
	Taxifolin	In silico	↓M^pro^	[253]
Flavonoid	Cyanidin	In silico	↓ACE2 and RdRp	[290]
	Kazinol A	In vitro	↓SARS-CoV 3CL^pro^ and PL^pro^	[283]
	Narcissin	In silico	Bind to ACE2	[289]
	Tomentin A-E	In silico	↓PL^pro^ in COVID-19	[287]
Flavone	Baicalin	In silico	↓TMPRSS2 and lead to inhibition of COVID-19	[204]
	Chrysin	In silico	↓ACE2 and decline neurological manifestation in COVID-19	[288]
Flavonol	Fisetin	In vitro, In silico	↓ACE2, ↓TNF-α, IL-6, IL-1β, ↑Nrf2, GPx, SOD	[282]
	Hesperetin	In vitro	↓ACE2 and reduce neurological sign in COVID-19	[291]
	Hesperetin	In vitro	↓ACE2 and reduce neurological sign in COVID-19	[291]
	Hyperin	In vitro	↓TNF-α, IL-6, IL-1β, NF-κB	[296]
Isoflavone	Daidzein	In vitro	↓TLR4, MyD88, NF-κB, MPO, IL-6, TNF-α	[294]
Polyphenol	Catechin	In silico	↓Spike protein, ↓viral entry, ↓ACE2	[243]
	Curcumin	In silico	↓spike protein, ↓viral entry, ↓ACE2 ↓TNF-α, IL-1β, IL-6, IL-18, NF-κB, COX-2	[242,243,301]
	Ellagic acid	In vitro	↓M^pro^, ↓replication	[302]
	Resveratrol	In vitro	↓SARS-CoV-2 infection.	[258,301]
	Sinigrin	In vitro	↓SARS-CoV 3CL^pro^	[284]
Terpenoid	Carvacrol	In silico	↓Spike protein	[292]
	Geraniol	In vitro	↓Spike protein, ↓TNF-α, IL-1β, IL-6, iNOS, COX-2	[292]
	Limonin	In silico	↓ACE2, 3CL^pro^, PL^pro^, RdRp and spike protein	[280]
	Thymol	In vitro	↓NF-κB, IL-6, TNF-α, IL-1β, ↑SOD	[295]
